# The complete chloroplast genome of an ornamental orchid, *Vanda coerulescens* (Orchidaceae)

**DOI:** 10.1080/23802359.2019.1704195

**Published:** 2020-01-10

**Authors:** Xiao-ting Wang, Ding-kun Liu, Wei-yao Zhu, Shu-zhao Zheng, Xiao-yun Yu, Ye Ai, Qing-hua Zhang

**Affiliations:** a College of Forestry, Fujian Agriculture and Forestry University, Fuzhou, China;; b College of Landscape Architecture, Fujian Agriculture and Forestry University, Fuzhou, China

**Keywords:** *Vanda coerulescens*, Orchidaceae, chloroplast genome, phylogenetic analysis

## Abstract

Though the chloroplast genomes of several *Vanda* species have been sequenced, there is little information about the complete chloroplast (cp) genome of *Vanda coerulescens*. Herein, we established the cp genome of *V. coerulescens*. The chloroplast genome circle was 149,410 bp in length, with the structure of an 85,954 bp large single-copy (LSC) region and a 11,526 bp small single-copy (SSC) region, which separated by two inverted repeat (IRs) regions of 25,965 bp. It encoded 130 genes, including 74 protein-coding genes, 38 tRNA genes and 8 rRNA genes. The overall GC-content of the whole plastome is 36.7%, whereas the corresponding values of the LSC, SSC, and IR regions ranged from 28.2% to 43.1%. In addition, the phylogenetic analysis base on 20 chloroplast genomes of Orchidaceae indicates that *V. brunnea* is closely related to *V. coerulescens*. This announcement of the complete *V. coerulescens* cp genome sequence could provide valuable information for further genetic modification and phylogenetic study in *Vanda* genus.

*Vanda* is a genus with magnificent flowers in the family Orchidaceae (Tanee et al. 2012) and has been widely used in commercial production. There are more than 70 species orchids in the *Vanda* genus (De et al. 2015; Zou et al. 2016). *Vanda coerulescens* is a high-value ornamental orchid and often used as a parent in breeding (Wang et al. 2013; Cao et al. 2014). *Vanda coerulescens* is found in the forest of Assam India, eastern Himalayas, Yunnan China, Myanamar and Thailand at elevations of 300–1600 m, and it grows on tree trunks (Chen and Alexandra 2009). There are many researches on the phylogenetic relationship and species identification of *Vanda* genus by using morphological observations and molecular phylogenetic analyses (Gardiner et al. 2013; De et al. 2015; Zou et al. 2016). Complete chloroplast (cp) genome information provide a valuable date for phylogenetic analysis. With the rapid development of high-throughput sequencing technology, many complete chloroplast genomes of *Vanda* genus have been sequenced (Ai et al. 2019; Chen et al. 2019; Zhou et al. 2019), but there is little information about the cp genome of *V. coerulescens*. In this study, we established the cp of *V. coerulescens* and confirmed its phylogenetic location. Our work will provide valuable information for further genetic modification and phylogenetic study in *Vanda* genus.

The samples of *V. coerulea* were collected from Mengla County, Xishuangbanna Dai Autonomous Prefecture, Yunnan Province, China (location: 21°26′4″N, 101°38′29″E), and the samples for experiments were preserved in the Herbarium of Fujian Agriculture and Forestry University with a voucher specimen code FAFU08139.

The total genomic DNA was extracted from fresh leaves by a plant DNA Kit (Omega, D3485), and sequenced by the BGISEQ-500 platform (BGI, Wuhan, China) (Mak et al. 2017). By using the fastq software (Chen et al. 2018), about 5 Gb clean reads were obtained after filtering out adapters and low-quality reads. Then, the processed data were used to assemble the complete chloroplast genome by GetOrganelle version1.5.2 (Jin et al. 2018) with the chloroplast genome of *V. brunnea* (NO. MK442937) as the reference sequence. After assessment of the assembled plastid genome, we annotated the new genome by using the Geneious R11.15 (Kearse et al. 2012). Finally, a complete chloroplast genome of *V. coerulescens* with annotation information was obtained and can be detected in GenBank with an accession number of MN711650.

The established circle chloroplast genome was 149,410 bp in length, including a small single-copy (SSC) region, a large single-copy (LSC) region and two inverted repeat (IR) regions in length of 11,526 bp, 85,954 bp and 25,965 bp, respectively. The overall GC-content of the whole plastome is 36.7%, whereas the corresponding values of the LSC, SSC, and IR regions were 33.9%, 28.2%, and 43.1%, respectively. It encoded 130 genes, including 74 protein-coding genes, 38 tRNA genes and 8 rRNA genes.

In addition, a phylogenetic analysis was carried out to investigate the phylogenetic location of *V. coerulescens* based on 19 reported chloroplast genomes of Orchidaceae species (Figure 1). All the sequences were downloaded from NCBI GenBank, the accession numbers were listed together with their species names to construct the phylogenetic tree. *Phalaenopsis equestris* and *Thrixspermum japonicum* were used as the outgroup species. HomBlock pipeline was adopted to align the 20 complete chloroplast genomes (Bi et al. 2018), and we checked the aligned file manually in Bioedit v5.0.9 (Hall 1999). Then, the RAxML-HPC program on CIPRES Science Gateway (https://www.phylo.org) was used to construct a maximum-likelihood (ML) tree with 1000 bootstrap replicates. The results showed that *V. coerulescens* is most closely related to *V. brunnea*, nested inside *Vanda* genus.

**Figure 1.**
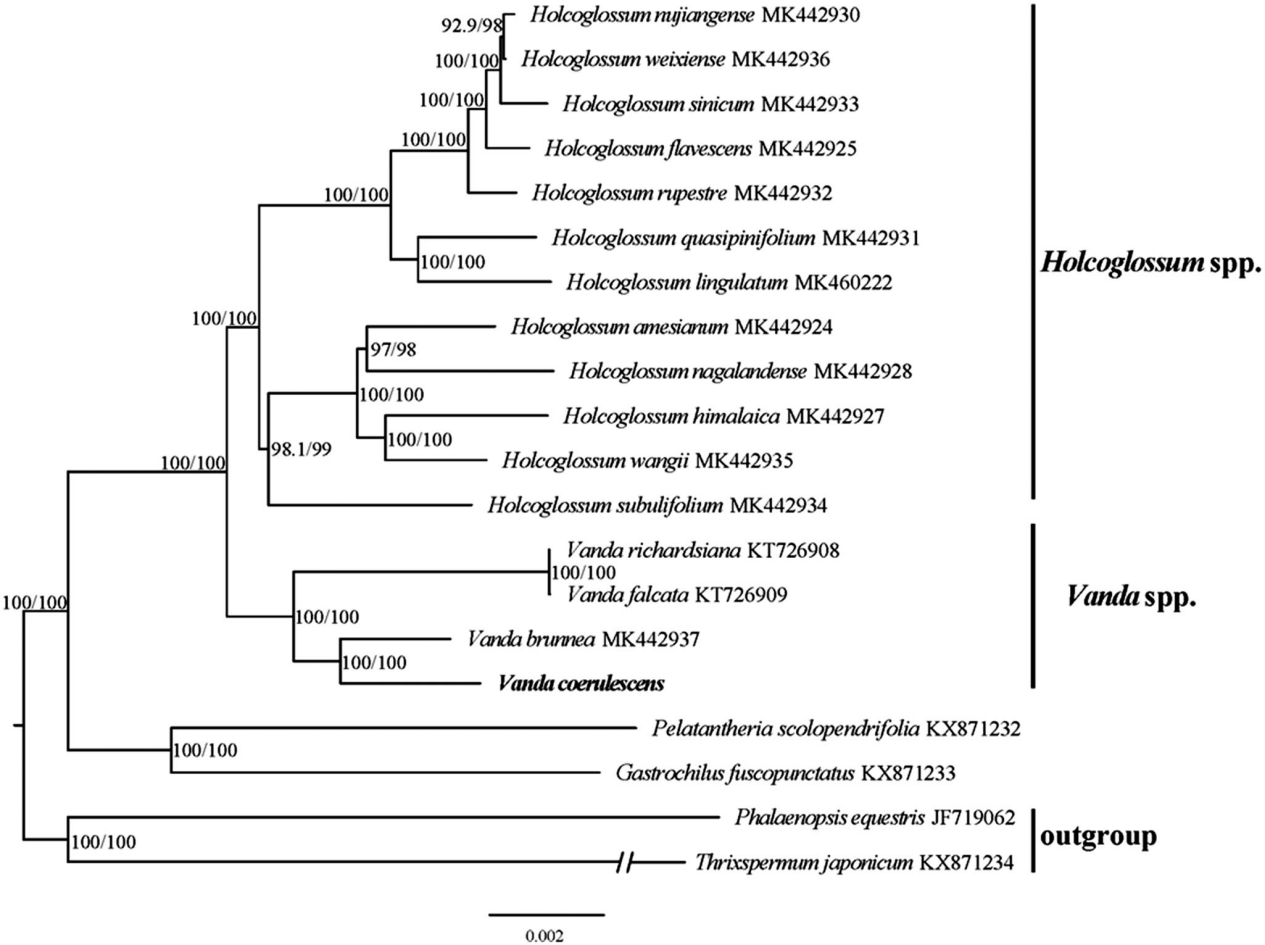
A phylogenetic tree was constructed based on 20 complete chloroplast genome sequences of Orchidaceae.
